# Green Carbon Dots as Additives of Biopolymer Films for Preserving from Oxidation of Oil-Based Products

**DOI:** 10.3390/antiox11112193

**Published:** 2022-11-05

**Authors:** Sandra Rodríguez-Varillas, Clarissa Murru, Marta Elena Díaz-García, Rosana Badía-Laíño

**Affiliations:** Department of Physical and Analytical Chemistry, Faculty of Chemistry, University of Oviedo, 33006 Asturias, Spain

**Keywords:** active packaging, composite films, nano-additives, green carbon dots, antioxidant properties

## Abstract

The deterioration of oil-based products during processing, distribution and storage has a major negative impact on the industry from an economic point of view. The spoilage of oil is mainly due to its oxidation which can be triggered by various factors, such as UV light, heating or the presence of impurities that result in the formation of radical species. In this context, several packaging alternatives have recently been developed with the aim to protect and extend the shelf life of oil-based products. This work aimed to study the antioxidant properties of bio-polymer-based films (BPFs) obtained from high methoxylated pectin (HMP) and sodium caseinate (CAS) and enriched with different concentrations of green carbon dots (gCDs), 0.25%, 0.50 and 1% *w*/*w*, obtained from apple pomace (APCDs) and rosemary powder (RCDs). The resulting films (gCDs-BPFs) have shown that the presence of gCDs not only modified the surface roughness of the films, but also positively affected their antioxidant properties. The addition of gCDs enhanced the radical inhibiting capacity of the raw BPFs by 42 and 62% for the films containing 1% RCDs and 1% APCDs, respectively. As a proof of the concept, two oil samples (edible and cosmetic) were treated with the obtained antioxidant films, and the results demonstrated that in both types of samples the oxidation process was minimized during the five days of the experiment. These results are promising and suggest that the antioxidant bio-polymer-based films could be excellent candidates for further production of active packaging.

## 1. Introduction

Oxidation is a process common to all types of lipids and involves a series of chemical reactions that degrade their quality and intrinsic value. The deteriorative changes caused by oxidation include unpleasant flavors, reduction in nutritional value and health properties of edible oils and lipids, but also a decrease in the physical and chemical properties for those oils with technical and industrial machine applications (industrial lubricants, hydraulic oils, etc.), thus shortening, in all cases, the useful life of oils and lipids [1,2].

Oxidation of edible oils (as well as many cosmetic products containing vegetable essential oils) can occur through three mechanisms: autoxidation, photo-oxidation, and enzyme-catalyzed oxidation. Autoxidation is a chain process that involves, during the initiation step, the formation of alkyl radicals mediated by the activation of oxygen by an energy source (light or heat) and/or by a catalytic compound such as transition metals (e.g., iron). These alkyl radicals initiate lipid oxidation and, simultaneously, a propagation phase is started with the formation of highly reactive hydroperoxide type radicals, LOO^●^. The process continues until a final termination step is reached where stable non-radical compounds are formed. Photooxidation requires the presence of photosensitizers (e.g., chlorophyll, riboflavin) and light for the generation of singlet oxygen responsible for reaction with unsaturated fatty acids to produce hydroperoxides. Finally, enzymatic oxidation initiates by cleavage of unsaturated fatty acids by lipoxygenase, the main enzyme involved in the process. The active site of lipoxygenase contains iron in ferrous form, responsible to trigger the oxidation process forming alkyl radicals. The oxidation of oils and lipids is a complex one, and the main reactions and factors involved in each have been documented and reviewed in recent papers [3,4]. 

It is known that prolonged exposure to UV radiation can promote the oxidation of oil-based products and shorten their useful life [5]. Therefore, to address this problem, researchers are trying to design sustainable materials capable of UV blocking as well as biodegradable packaging. With this aim, there are numerous works focused on the development of new biodegradable biopolymers for packages which incorporate natural antioxidant extracts or bioactive compounds obtained mainly from agro-industrial wastes [6,7,8,9]. On the other hand, nanotechnology is a burgeoning field of study in the manufacture of nano-based active packages; consequently, edible coatings enriched with nanoparticles have been demonstrated to be more advantageous than conventional packaging materials to preserve food products [10,11]. Nanomaterials like ZnO, TiO_2_, CeO_2_, SiO_2_, and Cu nanoparticles are widely used in UV blocking applications [12,13,14]; however, the addition of carbon nanoparticles, such as carbon dots (CDs) as antioxidant agents, to such films and coatings is a more innovative approach to the manufacture of active films with antioxidant properties to increase not only products’ shelf life but also their quality, freshness and safety. Although most papers dealing with the synthesis of CDs claim their biocompatibility and non-toxic properties, their impact on human health and on bioma is still in its infancy [15]. Nanoparticles, as metal-based as carbon-based, can modify the physical and mechanical properties of packaging polymers by improving their strength, durability, flexibility, and/or gas-barrier properties [16,17,18,19,20]. Proteins and polysaccharides have been widely used in the food industry as starting materials for the fabrication of films and coatings, [21,22,23]. Among natural materials, protein-polysaccharide based biopolymers reinforced with carbon nanomaterials have demonstrated to be promising for developing new biodegradable and edible wraps depending on their formulations [24,25,26,27,28]. 

Herein, high methoxylated pectin (HMP) and sodium caseinate (CAS) biopolymer films (BPFs) were produced based on a previous study by M. Jahromi et al. [29]. In this study, we combined the production of the films with the addition of green carbon dots (gCDs) obtained from rosemary (RCDs) and apple pomace (APCDs) to potentiate the valorization and use of industrial waste by-products. To this end, the synthesis, and the antioxidant activity of both types of gCDs and the corresponding gCD-BPFs, were investigated by the peroxide value method and the radical DPPH assay. Moreover, the optical properties of the materials were evaluated by UV and fluorescence spectrophotometry. As a proof of the concept, the gCD-BPFs were assayed as protective materials to prevent the oxidation of two model products: olive oil and cosmetic oil. The results obtained were promising as the oxidation of samples treated with gCD-BPFs was clearly minimized, thus paving the way for the use of antioxidant gCDs as active additives in the fabrication of biofilms and/or coatings that can increase the useful life of the oil-based products during their storage.

## 2. Materials and Methods

### 2.1. Chemicals

Olive Oil of the brand “Hacendado” was obtained from a Spanish local supermarket (Mercadona, Oviedo, Spain). Cosmetic Oil “Johnson&Johnson” was obtained from the local market Alimerka (Oviedo, Spain). Rosemary powder was purchased from a local supermarket (Netto, Regensburg, Germany) and depectinized apple pomace, Herbavital F12, was supplied by Herbstreith & Fox GmbH & Co. KG Pektin-Fabriken (Neuenbürg, Germany). Sodium Caseinate powder (CAS), HPLC grade methanol, ethanol, and chloroform were supplied by Merck Co. (Darmstadt, Germany). High-methoxylated pectin powder (HMP) (GENU^®^ Pectin, 50–75% esterification) was obtained from CPKelco (Lille Skensved, Denmark) and Glycerol 99% (PlusOne Glycerol) was from GE Healthcare (Chicago, IL, USA). Glacial acetic acid (95%), potassium iodate and sodium thiosulfate were obtained from Merck Co. (Darmstadt, Germany). DPPH reactive was obtained from Sigma Aldrich (Madrid, Spain). All solutions were prepared using distilled water. 

### 2.2. Instrumentation

Carbon dots were characterized using UV–visible spectroscopy with a Lambda 900 UV/VIS/NIR spectrophotometer (Perkin Elmer Spain, Madrid, Spain), while photoluminescence emission spectra were recorded on a Varian Cary Eclipse spectrofluorometer from Agilent Technologies (Las Rozas, Madrid, Spain), equipped with a xenon flash lamp. The absorbance and fluorescent spectra of the films were collected by an absolute measurement system on an FS5 fluorometer (Edinburgh Instruments, Livingston, UK) equipped with an integrating sphere module and FluOracle^®^ software (Livingston, UK). The redox potential measurements were performed using a pH/ion-meter GLP 22 (Hach Lange Spain, S.L.U., Barcelona, Spain) with an accuracy of ± 0.2 mV. Surface characterization was carried out using High Resolution Scanning Probe Microscopy from Agilent Technologies (Las Rozas, Madrid, Spain) and WSxM software (Madrid, Spain) for data treatment. The zeta potential and the particle size distribution were measured at room temperature using a Zeta Potential Analyser (Zetasizer ZS 3600, Malvern Instruments, Malvern, UK) and Zetasizer software (Malvern, UK). The Zeta potential of water-diluted gCDs solution was evaluated after 10 min of sonication in an ultrasonic bath. All experiments were carried out in triplicate.

### 2.3. Preparation of Bio-Films Enriched with Antioxidant gCDs

The synthesis of the gCDs by a hydrothermal method and the fabrication of enriched bio-films were carried out following the general procedure described by Jing et al. [30] without introducing significant modifications: 20 g of Rosemary (or Apple Pomace) were placed in a Pyrex glass beaker, heated in an oven at 200 °C (5 h along which 5 mL of distilled water were added to prevent the product from scorching every 30 min), and finally cooled to room temperature. Then, 20 mL of distilled water were added to the obtained black powders and centrifuged at 12,000 rpm for 50 min. Any remnant solid was removed by filtration through a 0.40 µm pore paper filter and the gCDs suspension was purified using a continuous nanofiltration system (MWCO 5 kDa) for 1 h. Finally, the suspension was lyophilized and the obtained gCDs were stored in the dark in a fridge until further use. For the preparation gCDs-BPFs, 25 mL of a sodium caseinate suspension (2% *v*/*v* CAS) and 75 mL of a high-methoxylated pectin suspension (2% *v*/*v* HMP) were mixed adding different concentrations of gCDs as active agents (0.25%, 0.5% and 1% *v*/*v*), using glycerol as plasticizer (0.75% *w*/*w*). Each mixture was poured into a plastic Petri dish, dried for 24 h at 40 °C and finally stored in a 53% relative humidity atmosphere (saturated magnesium nitrate solution) at room temperature. 

### 2.4. AFM-Based Surface Characterization

Atomic Force Microscopy (AFM) analyses were performed using a 2 cm × 2 cm piece of gCD-BPFs using non-contact mode. Roughness parameters obtained included peak-to-peak intensity values (p-p), average roughness (Ra) and root mean square roughness (Rq). Peak-to-peak distance refers to the difference between the lowest and the highest values measured in the sample area studied. Ra is the mean of the absolute values between the detected surface and the peaks while Rq is the square root of the mean of all values after squaring them [31]. All experiments were carried out in triplicate.

### 2.5. DPPH Free Radical Scavenging Assay

The DPPH free radical scavenging assay for the determination of the antioxidant activity of gCDs was realized according to the following protocol: a methanol solution of DPPH (100 µM) was prepared, and 1 mL was added to a given volume of the different gCDs dispersions. The mixture was stirred, and the reaction was left to take place in darkness for 30 min. The absorbance at 517 nm was finally measured to determine the concentration of the remaining free DPPH• radical. For the study of the antioxidant activity of the gCDs-BPFs with 1% RCD, 5 mg of the film (about 0.20 cm^2^) were placed in Eppendorf tubes and 1.5 mL of methanol and 0.5 mL of DPPH reagent were added. The tubes were covered with foil to allow the reaction to take place in darkness. All tests were performed in triplicate and methanol was used as blank control. The scavenging efficiency was calculated using Equation (1):

*DPPH inhibition* % = (1 − *As*/*Ab*) × 100 (1)

where *Ab* is the absorbance of the control and *As* is the absorbance of the sample.

### 2.6. Study of Olive Oil Oxidation in Presence of RCD-BPFs

A 2 cm × 2 cm piece of RCD-BPF was introduced into a 50 mL glass beaker with approximately 41 mm diameter containing 25 g of olive oil and was in contact with gentle stirring for 15 days at 25 °C (samples). Another beaker containing the same amount of oil in the absence of the biofilm sample was subjected to the same treatment and was used as a reference (blank). Every day, 0.5 g of the oils (blank and sample) were taken and dissolved in 5 mL of chloroform to measure the fluorescence signal at 348 nm, corresponding to the band emission of the RCDs, and at 322 nm, corresponding to the band emission of oil. All experiments were carried out in triplicate.

### 2.7. Olive and Cosmetic Oils Peroxide Value Determination

Peroxide Value method (PV) allowed to determine the iodine released by the oxidation of peroxides in the presence of potassium iodide. The iodine was volumetrically analyzed using a standard solution of sodium thiosulfate and the final point was potentiometrically determined using a pH/ion-meter equipped with a combined Pt/Ag/AgCl indicator electrode and a AgCl as the reference electrode. The thiosulfate solution was added using a burette. The PV of oil samples treated with gCD-BPFs was performed according to the following procedure: 36 glass tubes were filled with olive oil and placed in an oven, half of them containing a sample of a film with a size of 2 cm × 2 cm and a weight of 0.2 g. The remaining tubes were used as blank. The samples were heated for 5 days at 40 °C to allow a slow formation of reactive radical species. To evaluate the PV, 10 g of each oil sample was weighed and dissolved in 50 mL of a mixture of Cl_3_CH/glacial acetic acid (3:2 *v*/*v*). Then, 0.5 mL of a saturated solution of KI aqueous solution was added. The mixture was gently stirred up to the finish of the analysis. Measurements were performed after each addition of 0.0050 M sodium thiosulfate. Similar operations were performed for the blank solution. The PV was calculated using Equation (2):

(2) $$PV=\frac{\left(V-V_{0}\right)\times C_{t}\times 1000}{w}$$

where *V* and *V_0_* were the volumes of the sodium thiosulfate standard solution added at the final point to the analyzed oil sample and blank solution, respectively. *C_t_* is the molar concentration of the standard solution of sodium thiosulfate and *w* is the weight (g) of the oil sample. A parallel study was carried out using cosmetic oil and following the same procedure explained above but heating the samples at 70 °C.

## 3. Results and Discussion

**Figure 1:**
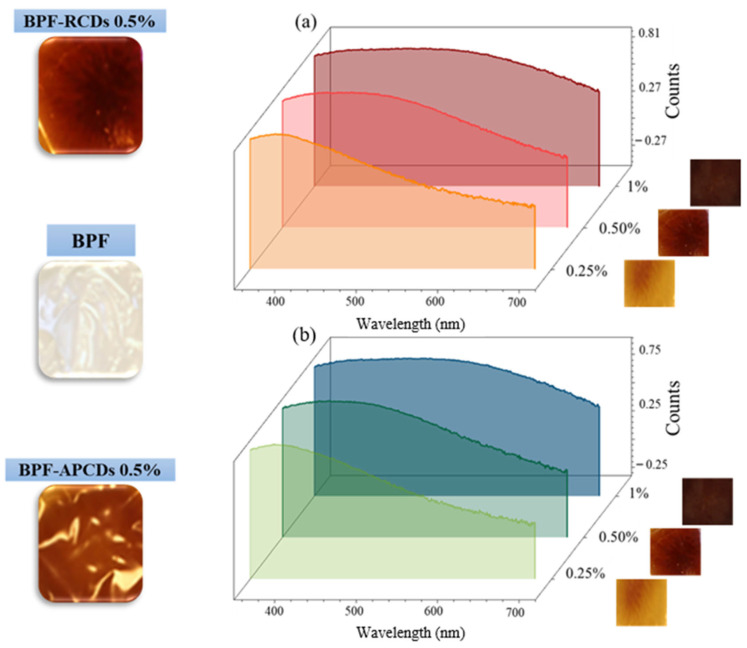
Absorption spectra and photographs of BPF and gCDs-BPFs: (**a**) APCDs, (**b**) RCDs.

Optical, morphological and antioxidant properties of the gCD-BPFs were studied to evaluate their potential as active protection of oil base products. The different BPFs obtained were shown to have a homogeneous structure (See the photographs inserted in Figure 1 and manageability.

### 3.1. Optical Characterization of gCDs and gCD-BPFs

**Figure 2:**
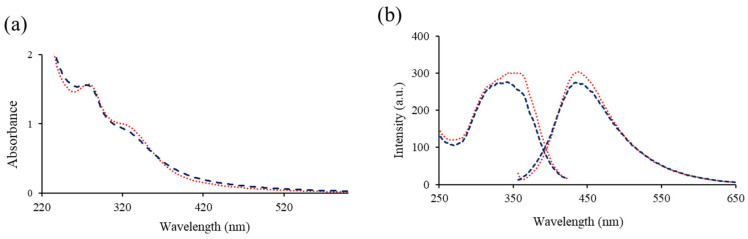
(**a**) Absorption and (**b**) excitation and emission spectra of gCDs: APCDs (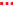); RCDs (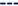).

In a previous work [17] we could determine that the RCDs and APCDs were approximately spherical shape with an average diameter of 3.8 ± 0.75 nm for RCDs and 4.2 ± 0.94 nm for APCDs. The UV absorption spectra of RCDs and APCDs (Figure 2a) showed similar spectral characteristics, with a band at 280 nm corresponding to π → π * transitions due to sp^2^ hybridized groups (C-C double bonds) and another band at 330 nm assigned to n → π * transitions due to C=O groups, C-N or C-OH in sp^3^ hybridized bonds. There are no significant differences in the photoluminescence spectra of both types of gCDs: an emission band with a maximum intensity at 440 nm and an excitation band with a maximum intensity at 350 nm (Figure 2b).

**Figure 3:**
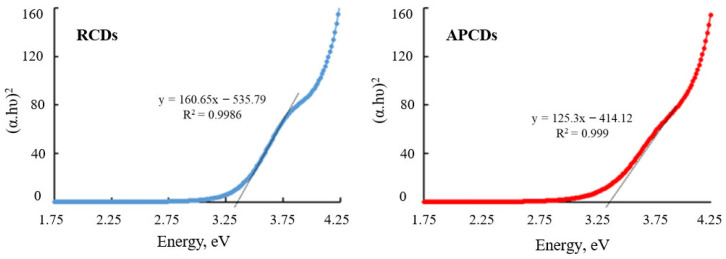
Tauc plots for estimation of the gCDs bandgap.

Starting from the absorption spectra it was also possible to estimate the band gap (*E_g_*) for the gCDs using Tauc’s law for direct transitions (Equation (3)) [32]:

(3) $${\left(\alpha h\nu \right)}^{2}=k\left(h\nu -E_{g}\right)$$

where *α* is the absorption coefficient, *hν* is the energy of the incident light and *k* is a constant. *E_g_* was estimated at the intersection point of the extrapolated straight line with the abscissa axis from the graphical representation of *(αhν)^2^* vs. *hν* (Figure 3). The band gap of gCDs was found to be 3.34 and 3.30 eV for RCDs and APCDs, respectively. These values are consistent with those in works already published [33,34,35,36]. The meaning of these data concerning the reactivity of the gCDs [37] in the context of the present paper has been discussed in detail in the Supplementary Information.

As can be observed in Figure 1, the RCD-BPFs with a low RCDs content (0.25%) showed a well-defined absorption band with a maximum at 420 nm, while those with a higher concentration presented broad and poorly defined absorption bands, probably due to internal filter effect. This phenomenon could be a factor to take into account when considering the possible practical applications of this type of BPF since those with the highest concentration of gCDs (0.5 and 1.0%) could act as a screen for external radiation if they were used to wrap light sensitive products (e.g., oils that oxidize and produce free radicals).

**Figure 4:**
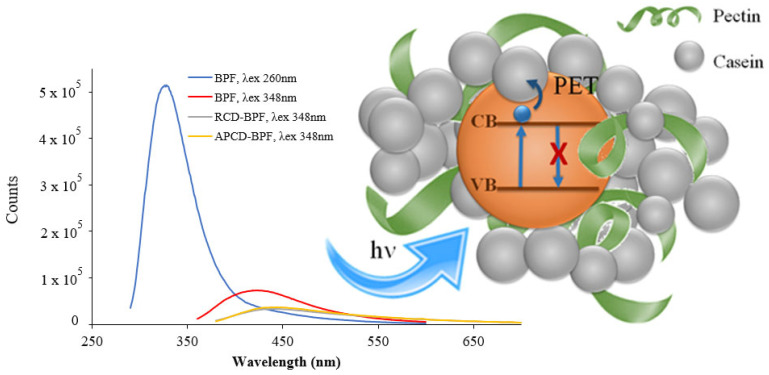
Fluorescence emission spectra of BPF (blue λex = 260 nm, red, λex = 348 nm) and gCD-BPFs containing 0.25% RCDs (gray, λex = 348 nm) and 0.25% APCDs (orange, λex = 348 nm). Inset: gCD-BPFs fluorescence deactivation through a PET process.

To determine the photoluminescent properties of the films, several samples of the same size (1 cm × 1 cm) were prepared and placed on top of the front-face support to record the excitation and fluorescence spectra. BPFs in the absence of gCDs were used as reference blanks. The fluorescence spectra of the films were also recorded, as seen in Figure 4. Using an excitation wavelength at 260 nm, the BPF without gCDs presented an intense fluorescence band with a maximum intensity at 325 nm, ascribed to the intrinsic fluorescence of casein [38]. BPF excitation at 348 nm, the maximum of the excitation band of gCDs, produced only a weak emission band at 425 nm. It was observed also that gCD-BPFs, when excited at 348 nm, also presented very weak emission bands at 438 nm despite the presence of gCDs. This fact could be explained by the presence of two fluorescent systems in gCD-BPFs that interact through a deactivation redox process: photoinduced electron transfer (PET). The possible mechanism for PET is proposed in the scheme inserted in Figure 4: when the gCDs were excited at their wavelength of excitation maximum, an electron was promoted from the valence band to the conduction band. In the nano-environment surrounding the gCDs, the electron may be transferred to casein and/or pectin, both materials with antioxidant capacity [39,40], thus preventing the electron its return to the valence band and, therefore, the fluorescence emission of the gCDs-BPFs seriously decreased. This process has been explained in terms of HOMO-LUMO energies [20,41,42] (Supplementary Information).

#### Functional Characterization of gCDs and gCD-BPFs

**Figure 5:**
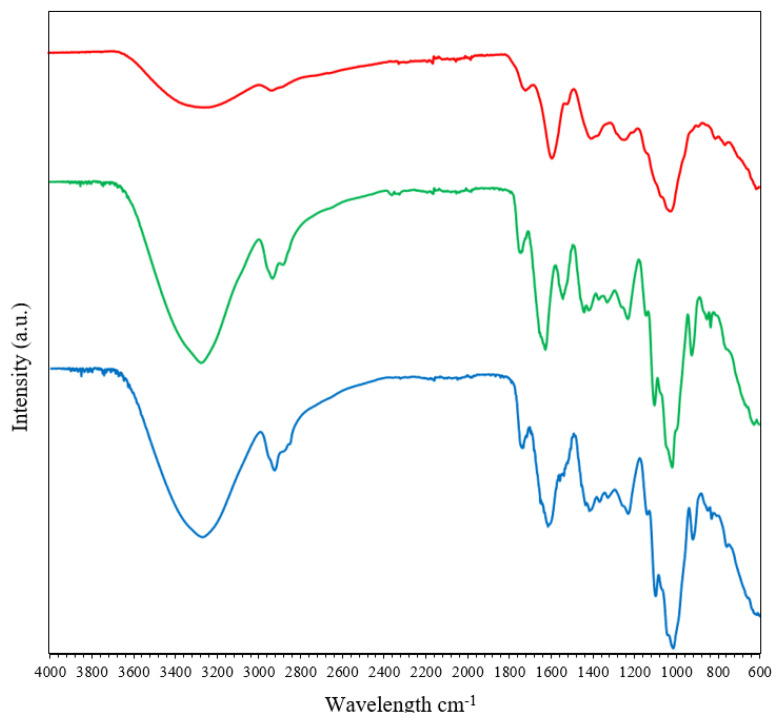
ATR-FTIR spectra of RCDs (▬); BPF (▬) and 1% RCD-BPF (▬).

In order to obtain a characterization of functional groups in the nanomaterials, ATR-FTIR spectra were obtained. As can be seen in Figure 5, the region with the most information on the FTIR spectra BPFs and gCD-BPFs is the one that ranges between 900 and 1800 cm^−1^. This region is important for defining the degree of esterification of pectin: the bands at 1735 cm^−1^ and 1612 cm^−1^ can be assigned to the stretching of the C=O in the methyl-ester group and to the asymmetric stretching of the carbonyl group in the unesterified carboxylate of pectin [43], respectively. Other detected bands related to the structural conformation of glycosidic bonds in pectin were those observed in the 1100–920 cm^−1^ region: the band at 1140 cm^−1^ is characteristic of O-C-O asymmetric stretching, while the band at 1020 cm^−1^ is assigned C–O stretching and C–C stretching [44]. The FTIR spectrum of RCDs presented a broad band at 3200–3600 cm^−1^ attributed to stretching vibrations of -O-H bonds and N-H bonds. The bands at 1590 cm^−1^, 1014 cm^−1^ and 1700 cm^−1^ were assigned to the absorption of superficial carboxylic groups, due to vibrations of C-O bonds present in polysaccharides and to simple ketones and carboxylic groups, respectively. The band at 1520 cm^−1^ observed in BPFs was assigned to amide II vibrations (stretching vibrations of CN bonds in combination with bending vibrations of NH bonds) of casein [39]. This band has practically disappeared in the gCD-BPFs spectrum. On the other hand, the band observed at 1612 cm^−1^ BPFs has shifted to 1590 cm^−1^ in the gCD-BPFs spectrum, probably by interaction with the RCDs of casein and pectin, respectively. The relative intensity of the film bands did not change with the presence of the RCDs. The ATR-FTIR spectra of the APCDs and 1% APCD-BPFs are shown in Figure S1 (Supplementary Materials), exhibiting a similar pattern to the RCD-based systems.

### 3.2. Surface Characterization and Colloidal Stability of CDs

**Figure 6:**
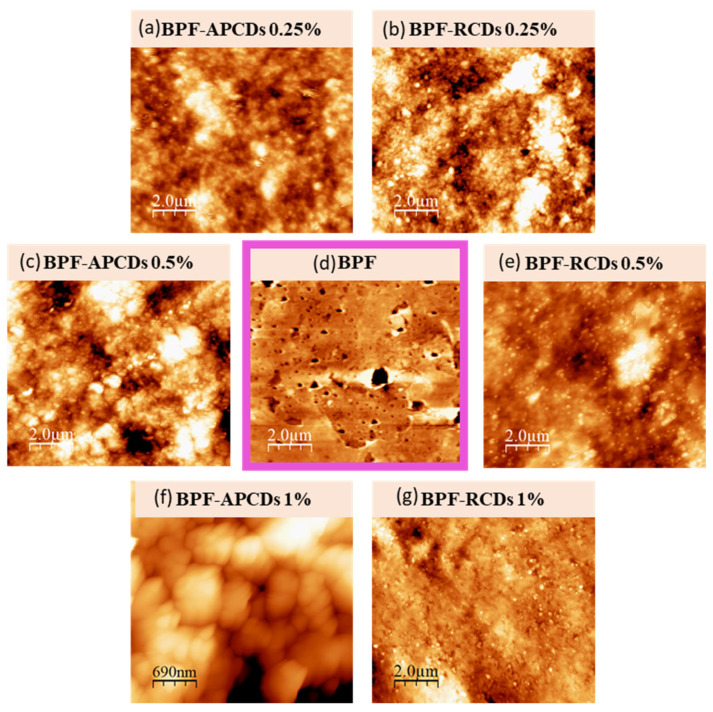
Surface images of: biofilms reinforced with (**a**) 0.25 %, (**c**) 0.5 % and (**f**) 1 % of APCDs; (**d**) biofilm without gCDs, and biofilms reinforced with (**b**) 0.25 %, (**e**) 0.5 % and (**g**) 1 % of RCDs.

In order to obtain a morphological characterization of the surface, studies using AFM and DLS were carried out. Based on the AFM images (Figure 6), the roughness parameters of BPFs changed when the gCDs were added and these variations depended on the type of gCDs used and their concentration. The presence of gCDs into the BPFs matrix seemed to modify their structure, filling the pores initially observed in BPFs (Figure 6d). The results obtained for average roughness (Ra), root mean square roughness (Rq) and peak-to-peak intensity values (p-p) for each sample are collected in Table 1. In comparison with the neat BPF, the roughness Ra values were lower in RCD-BPFs, independently of their concentration. These results could be explained by the RCDs filling up the BPF pores, thus resulting in a smoother BPF surface.

However, for APCD-BPFs the value of Ra only decreased for an APCDs concentration of 0.25%, while higher concentrations raised the Ra, particularly for the 1% APCD-BPF. This effect could be ascribed to the formation of agglomerates (see magnification Figure 6f).

ELS measurements provide information about the surface charge of nanoparticles. The zeta potential (ζ) is the potential established between medium and charged nanoparticles in an aqueous solution, and it is related to colloidal stability [45]: particles with large ζ-potential (>±30 mV) means strong repulsive forces that avoid the particles to aggregate (stable dispersion), while low ζ-potential values (<±10 mV) indicate that dispersions are highly unstable, showing flocculation processes. In this study, the experimental values of ζ-potential for APCDs and RCDs were −4 ± 1 mV and −13 ± 2 mV, respectively. These results suggested that the RCDs suspensions were more stable than those of APCDs which, in turn, support the formation of aggregates observed in AFM images when 0.5 and 1% APCDs were added to the BPFs. Consequently, the addition of RCDs (even at high contents as 1%) to BPFs resulted in homogeneous and less rough films because coagulation and flocculation phenomena did not manifest during the formation of the RCD-BPFs. Furthermore, zeta potential values provided information about the electrical charge on the nanoparticle surface. Negative values obtained in both, RCDs and APCDs, demonstrate that their surface is negatively charged due to the presence of carboxylic acid and hydroxyl groups [46]. The more positive ζ-potential of APCDs could be explained by the probable presence of residual amino components (e.g., proteins, vitamins, etc.) [47] that may impart some positive charges to the synthesized APCDs.

### 3.3. Antioxidant Activity of gCDs and gCD-BPFs

Antioxidants interfere with the action of free radicals by intervening at any of the three major steps of the free radical-mediated oxidative process, viz., initiation, propagation and/or termination. DPPH assay allows for evaluation of the antioxidant activity due to the presence of an odd electron on the nitrogen atom in the DPPH molecule, forming the DPPH•. This radical can be reduced to DPPH-H by attaching an electron or a hydrogen atom. The purple colour of an ethanol DPPH• solution (absorbance at 517 nm) changes to yellow as the electron pairs off. The colour change at 517 nm is used to calculate the radical scavenging activity (% of inhibition) using Equation (1).

**Figure 7:**
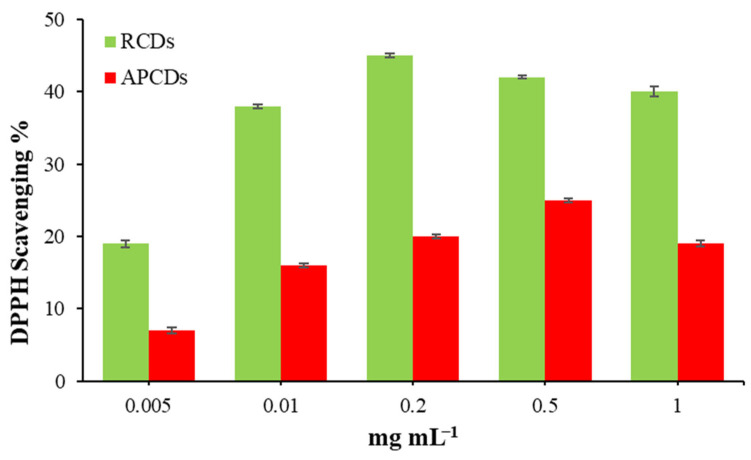
Antioxidant activity of RCDs and APCDs using the DPPH assay.

**Figure 8:**
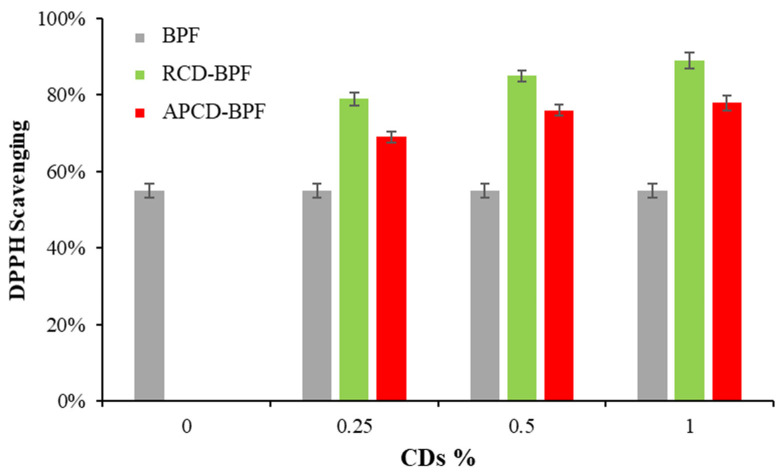
Antioxidant activity of gCDs-BPFs.

The results of the DPPH assay to determine the antioxidant activity of the gCDs in solution are shown in Figure 7. It can be observed that the DPPH inhibition activity of gCDs depended on their concentration. For RCDs, it was observed that the percentage of inhibition of the DPPH• increases up to a concentration of 0.2 mg/mL, over which a slight decrease was observed. In the case of APCDs, a steady growth in the percentage of inhibition of DPPH• was also observed up to a concentration of 0.5% mg/mL. For the same concentration of gCDs used, the RCDs present a higher activity; so, at a concentration of 0.2 mg/mL, the percentage of inhibition DPPH• by RCDs and APCDs was found to be 45% and 20%, respectively. Figure 8 shows the results obtained for the inhibition assays of gCD-BPFs as a function of the concentration of CDs used in their preparation. It is important to note that neat BPFs exhibit a relatively high antioxidant activity itself, with a 55% inhibition of DPPH• under the experimental conditions used. This fact could be attributed to the antioxidant activity of casein [39] and pectin [48]. It has been demonstrated that the presence of aromatic amino acids such as Trp, Tyr and Phe favour the antioxidant activity of peptides and that the indole and pyrrolidine rings in Trp and Pro, respectively, can act as hydrogen donors (via hydroxyl groups) in the deactivation of hydroxyl radicals [40,49]. It has also been studied that pectin has antioxidant properties due to contaminants (e.g., phenols) that are co-extracted with pectin [50]. The addition of gCDs notably increases the radical inhibiting capacity of the films: 62% for 1% RCD-BPFs and 42% for 1% APCD-BPFs.

### 3.4. Study of the Stability of gCD-BPFs in Oily Medium

**Figure 9:**
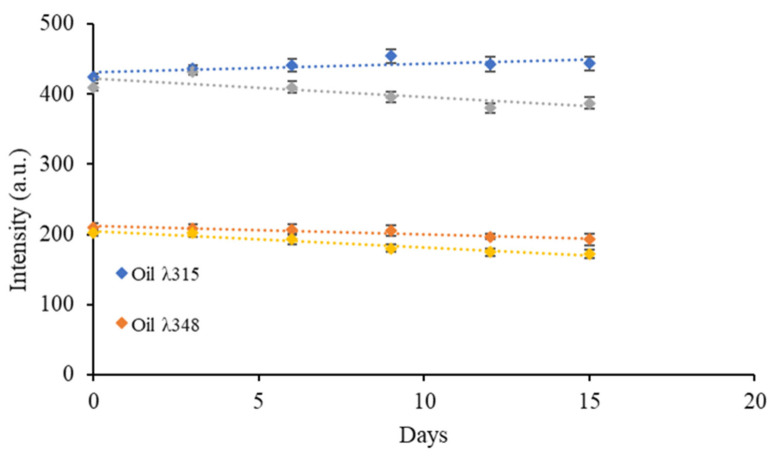
Study of the stability of RCDs contained in RCD-BPFs in contact with olive oil.

Since the objective of developing gCDs-BPFs was their final use as a material to produce active packaging, it was necessary to investigate their stability with respect to the leaching of the gCDs from the films towards the oily sample. Given the intrinsic fluorescence presented by gCDs dispersed in oils, the study was carried out by immersing a 2 cm × 2 cm piece of the 1% RCD-BPFs in a sample of 25 mL of commercial olive oil, with constant gently stirring during 15 days. The fluorescence intensity of the oil at the maximum excitation wavelength of the RCDs was measured every three days. Figure 9 shows the variation in the fluorescence signal along the time assayed. It was possible to observe that the emission intensity of the oil, with an emission maximum at 322 nm, was slightly higher than the oil containing the RCD-BPFs, and it remained statistically constant over time. However, the emission of the oil containing the 1% RCD-BPFs slightly, which seemed to indicate that the RCDs were not released from the film due to the hydrophobic nature of the medium and the low dispersibility of the nanoparticles in it. In Figure 9, it can also be observed that at 348 nm (maximum emission wavelength of RCDs), the trend followed a similar pattern although the difference in emission intensities between the neat oil and the oil containing the 1% RCD-BPFs was smaller. A plausible explanation for the decrease in fluorescence intensity would be related to the presence of water/humidity in the oil containing the 1% RCD-BPFs: the films were stored under a relative humidity of 57% and it was plausible that during the test, low traces of water were released forming reverse micelles modifying the polarity of the medium [4]. A parallel study was carried out using cosmetic oil and the results, similar to those described for olive oil, are collected in Figure S2 (Supplementary Materials).

### 3.5. Proof of Concept

#### 3.5.1. Influence of CD-BPFs on the Peroxide Value of Edible and Cosmetic Oils

**Figure 10:**
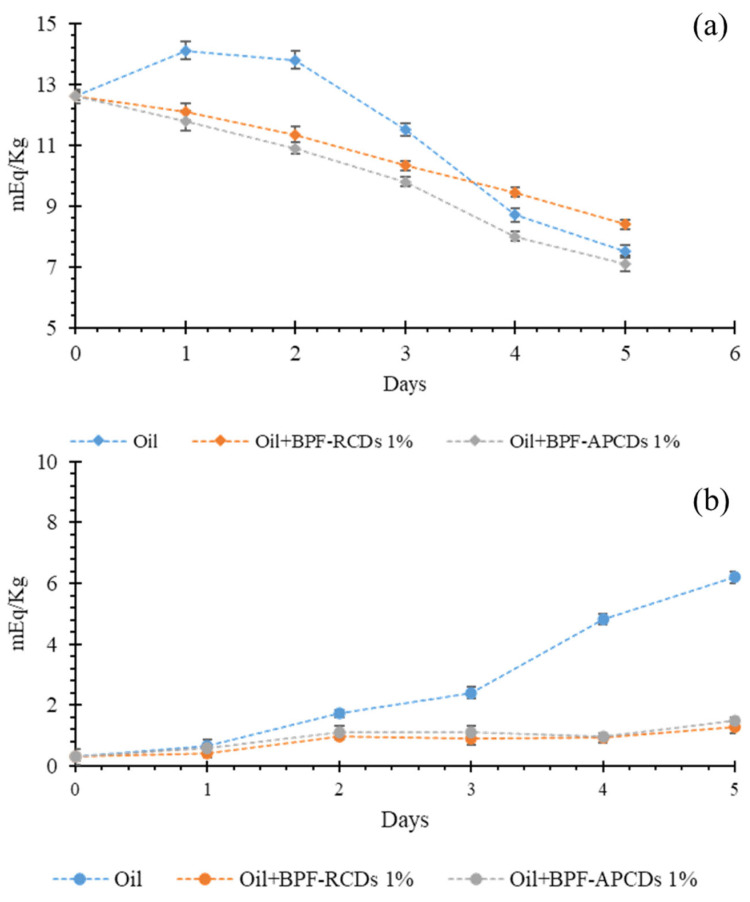
Study of the formation of radical species in samples of olive oil (**a**) and cosmetic oil (**b**) treated with antioxidant 1% gCD-BPFs.

Over the years, different methods have been developed to determine the degree of oxidation of oils (and fats), both for industrial and human or animal consumption. Among these methods, the Peroxide Value (PV) is one of the main indicators used to evaluate the formation of hydroperoxides. PV is an indicator used to evaluate the amount of hydroperoxides formed in terms of milliequivalents per g of oil, which oxidize potassium iodide under the standard conditions of the text [51]. In order to verify the efficacy of the gCDs-BPFs to decrease and/or to stop the initial oxidation process, the PV of the oils (edible and cosmetic) was determined in the presence and absence of gCDs-BPFs using the iodometric titration by potentiometric determination of the endpoint by signing the protocol described in the Materials and Methods section. To carry out this experiment, only the films that contained a higher percentage of gCDs (1% gCD-BPFs) were used, as they turned out to have the highest antioxidant potential as demonstrated by the DPPH test. The results are collected in Figure 10 where it can be observed that the most significant changes in PV took place during the first days of the experiment. In the absence of films, there was an increase in PV during the first 2 days due to the oxidation of the oil, followed by a rapid decrease in PV, probably due to the termination of the free radical chain reactions. Similar behavior was observed in the measurements of the cosmetic oil samples, although the initial PV was significantly lower than in olive oil. Still, it rose continuously during the time tested. Samples containing 1% gCD-BPFs have shown that the PV in olive oil began to decrease from day one, suggesting that the films deactivated the peroxides generated in these early stages of the oxidation process. In cosmetic oil, the PV remained at a low value throughout the study time, indicating that the 1% gCD-BPFs stabilized the oil due to the consumption of the peroxides generated.

#### 3.5.2. Potential Applications of Bio-Polymer Films in Wet Food Samples

**Figure 11:**
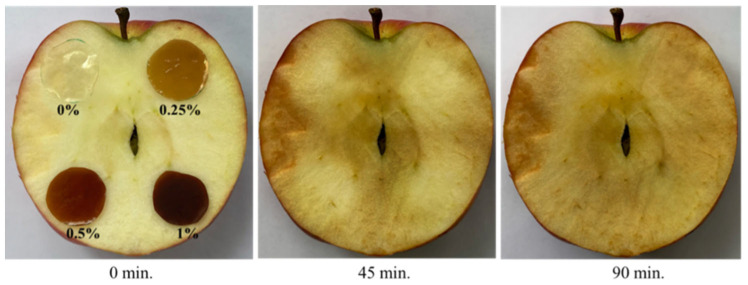
Oxidation of an apple treated with BPF and gCD-BPFs.

In order to check whether the gCD-BPFs developed offered practical potential use as antioxidant wrappers in products with a high humidity, apples were selected as they oxidize very quickly. Four bio-polymer films were tested: BPF, gCD-BPFs 0.25%, gCD-BPFs 0.5%, and gCD-BPFs 1%. The films were cut into similarly sized circular sections and placed on the surface of half apples, as shown in Figure 11. The samples were kept at room temperature of 25 °C for 90 min. The films were then gently removed after 45 min, and it was observed that those areas where they were placed were paler than the rest of the fruit surface. Removal of the gCD-BPFs from the apple surface was difficult due to their hydrophilicity. Once the films were removed, the apples were allowed to continue to oxidize for another 45 min and it could be observed that those zones previously covered with the gCD-BPFs continued being oxidized, indicating that the gCDs were not released on the surface of the wet fruit. At first glance, it was not possible to assess the contribution that the presence and/or nature of gCDs had on this behaviour. Likewise, the problem of the stability of neat BPFs films in aqueous media is still to be solved [9,52]. However, the results allow foreseeing that the system could have a good performance in products with a high degree of humidity, once the stability is achieved.

## 4. Conclusions

In this work, the optical and antioxidant properties of gCDs obtained from rosemary leaves (RCDs) and apple pomace leaves (APCDs) have been investigated. Some gCDs have been used as additives for the fabrication of active biopolymer-based films, gCD-BPFs. Both types of gCDs have fluorescent and antioxidant properties, being the RCDs the ones with the highest antioxidant activity against the radical DPPH•. BPFs also showed antioxidant activity (due to casein and pectin), which increased with the addition of gCDs. Through AFM surface characterization it was observed that BPFs reinforced with RCDs were less rough than biofilms containing APCDs, as the latter showed a greater tendency to form aggregates at high concentrations as demonstrated by DLS measurements. It was possible to demonstrate, by determining the peroxide value, that gCD-BPFs decreased the oxidation process of edible and cosmetic oils. However, films in contact with edible products with high water content were destabilized due to their hydrophilicity. In order to expand the potential of BPFs and gCD-BPFs as active antioxidant materials as wrappers for products with high water content, it will be mandatory in the future to delve into their stabilization and optimization of physical properties.

## Figures and Tables

**Table 1:** Roughness parameters of BPFs with and without CDs reinforcement.

Sample	Ra (nm)	Rq (nm)	p-p (nm)
BPF	12.4	16.9	120.1
RCD-BPF 0.25%	10.6	13.0	62.2
RCD-BPF 0.5%	10.0	12.5	61.1
RCD-BPF 1%	8.1	10.2	50.0
APCD-BPF 0.25%	10.0	12.2	55.0
APCD-BPF 0.5%	14.0	17.2	77.0
APCD-BPF 1%	93.9	114.0	531.1

## Data Availability

Data is contained within the article.

## Supplementary Materials

The following supporting information can be downloaded at: https://www.mdpi.com/article/10.3390/antiox11112193/s1, Figure S1: ATR-FTIR spectra of gCDs, Figure S2: Study of the stability of RCDs contained in RCD-BPFs in contact with edible oil, Tauc plots: Implications of the RCDs band-gap value and HOMO-LUMO analysis.
