# Supplementary material for: Green Carbon Dots as Additives of Biopolymer Films for Preserving from Oxidation of Oil-Based Products
Source: Antioxidants (Basel). 2022 Nov 5;11(11):2193. doi: 10.3390/antiox11112193 (PMC9686731; doi:10.3390/antiox11112193)
Supplement: Supplementary file 1 [file antioxidants-11-02193-s001.zip › antioxidants-1991711-supplementary.pdf]

Supplementary Information

Green Carbon Dots as Additives of Biopolymer Films for Preserving from Oxidation of Oil-Based Products

Sandra Rodríguez-Varillas †, Clarissa Murru †, Marta Elena Díaz-García, Rosana Badía-Laíño

† Both authors contributed equally to this study.

Department of Physical and Analytical Chemistry, Faculty of Chemistry, University of Oviedo, 33006 Asturias, Spain

Correspondence: rbadia@uniovi.es

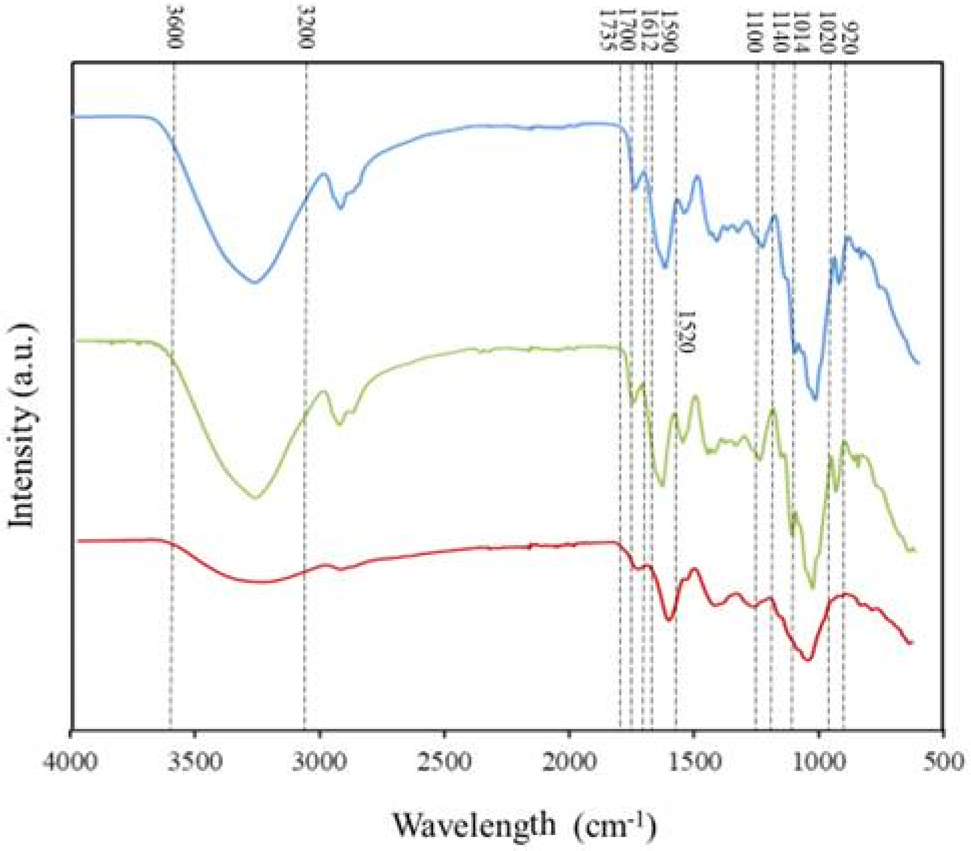

Figure S1. ATR-FTIR spectra of APCDs **(—);** BPF **(—)** and 1% APCDs -BPF **(—).**

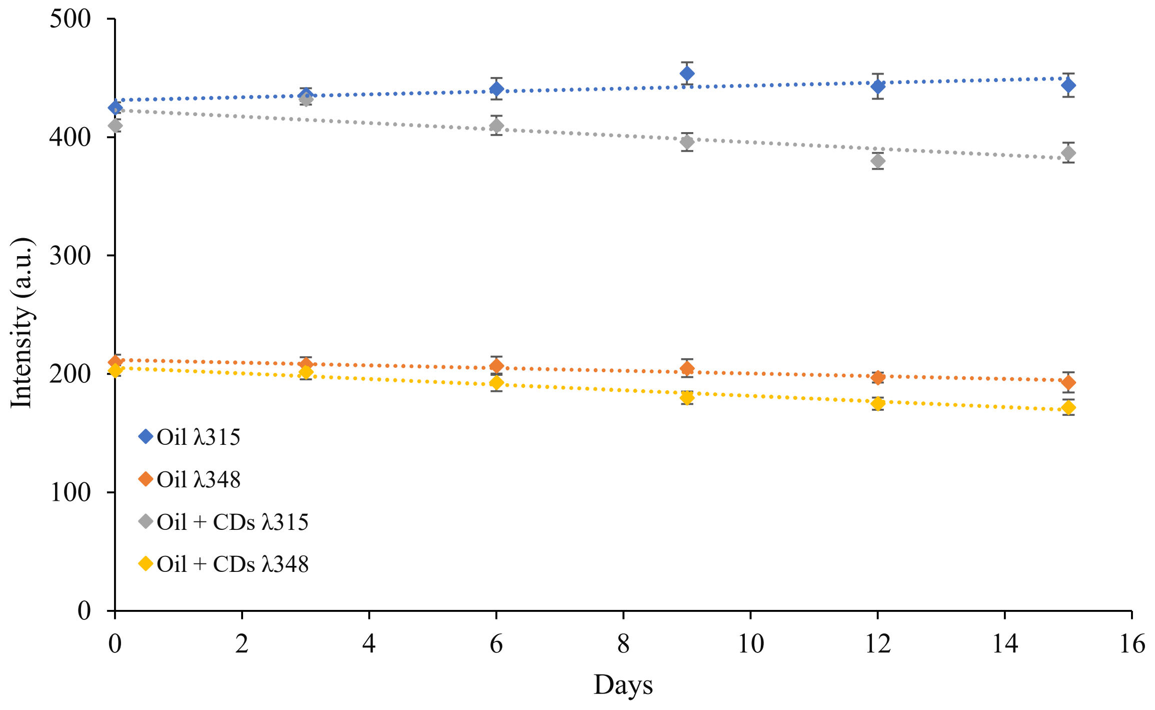

Figure S2. Study of the stability of RCDs contained in RCD-BPFs in contact with edible oil.

Tauc Plots: Implications of the RCDs Band-Gap Value and HOMO-LUMO Analysis.

Tauc plot allows to calculate the band-gap of semi-conductors and, in the nanoscale range, it gives precise information concerning the band structure of nanoparticles (NPs). It is well known, that the band gap represents the chemical reactivity and kinetic stability of the NPs [37]. For example, a large band-gap signifies that the NPs have a high-kinetic stability due to the energetically unfavourable electron promotion from the valence to the conduction band.

In the present manuscript, the relatively low band gaps of RCDs (3.3 eV) and APCDs (3.4 eV) means that these CDs are reactive enough to interact with the BPFs components as demonstrated by the results obtained by ATR-FTIR, spectrophotometric and fluorescence analyses described in the manuscript. Besides, the band-gap information is useful in determining the CDs charge transfer interactions within the BPFs (e.g. the PET process that resulted in fluorescence quenching of CDs).

As precise band gap data of biopolymers (e.g. proteins) are still lacking, in a first approximation we can suppose that RCDs interact with leucine as model a molecule for casein. Leucine content is highest in cow (108 ± 2.3 mg/g), camel (96 ± 2.2 mg/g) and buffalo (90 ± 2.4 mg/g) milk caseins.

The interaction of RCDs with the protein (and then the PET process) could be then described in terms of HOMO/LUMO energy values. Applying the mathematical formulations described in our previous paper [20], the calculated values of the energy of the conduction band (LUMO) of RCDs resulted to be 9.89 eV, while that of the valence band (HOMO) was 13.33 eV. On the other hand, leucine E_{HOMO} and E_{LUMO} have been reported to be −3.830 and 2.953 eV, respectively [41]. Upon excitation of the RCDs, the electron transfer to the LUMO orbital of leucine is energetically favoured and the RCDs fluorescence became off. (Figure S3)

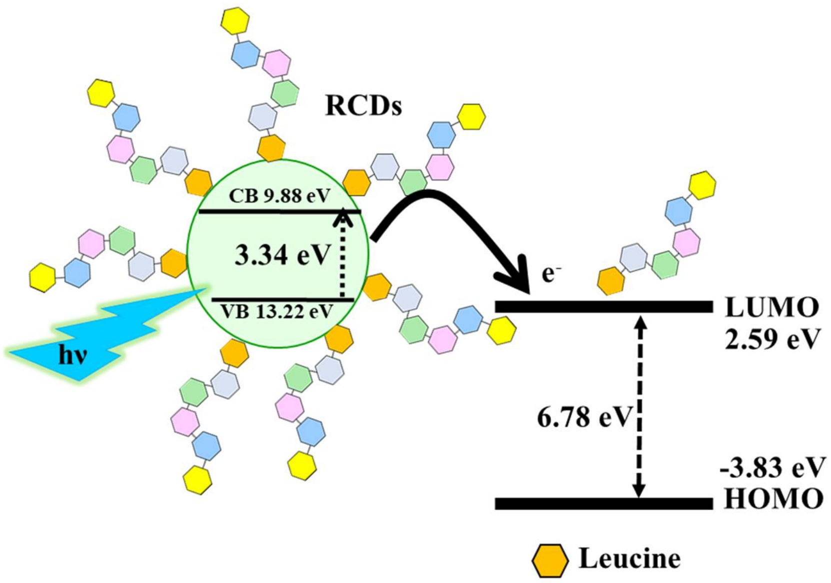

Figure S3. Pictorial representation of the PET process in terms of energy.

Similar considerations could apply if the pectin component of the BPF is considered: the E_{HOMO}, E_{LUMO} and band-gap for pectin have been reported to be −7.126, 0.077 and 7.204, respectively [42].
